# Supplementary material for: Metabolic Complementation in Bacterial Communities: Necessary Conditions and Optimality
Source: Front Microbiol. 2016 Oct 7;7:1553. doi: 10.3389/fmicb.2016.01553 (PMC5054487; doi:10.3389/fmicb.2016.01553)
Supplement: Supplementary file 3 [file Presentation1.PDF]

Supplementary Information

Article title: Metabolic complementation in endosymbiotic consortia: genome reduction and protein-associated costs

*Matteo Mori1[1,2], *Miguel Ponce-de-León[1], Juli Peretó[3], Francisco Montero[1]

1 - Departamento de Bioquímica y Biología Molecular I, Facultad de Ciencias Químicas, Universidad Complutense de Madrid, Madrid Spain

2 - Department of Physics, University of California at San Diego, La Jolla, CA, USA

3 - Departament of Biochemestry and Molecular Biology and Institut Cavanilles de Biodiversitat i Biologia Evolutiva, Universitat de València, Valencia Spain

Supplementary Note S1: Mathematical properties of the crossfeeding model and its optimization

The cross-feeding model we developed in the main text is a kinetic model in which the fluxes are explicitly modelled as a function of metabolite's and enzyme's concentrations. The optimization problem discussed in our work is the minimization of the total enzyme levels for the whole population considered, with constraints on the minimum production fluxes of the final product of the biosynthetic pathways. In this note we describe some useful mathematical properties of the model:

(**Section S1.1**) Consider a solution to the optimization problem, obtained using some demand fluxes $J_{P,i}$ and permeability constants D_α. Then, consider another problem in which the parameters $J'_{P,i}$ and D'_α are obtained by multiplying the previous ones by the same constant $a>0$, so that $J'_{P,i}=a J_{P,i}$ and $D'_\alpha = a D_\alpha$. Then, the solution to this new optimization problem is obtained from the old solution by multiplying the enzyme levels by the same constant, $[E]'_{\alpha,i}=a[E]_{\alpha,i}$. In particular, the two solutions have the same protein asymmetries $A_\alpha=([E]_{\alpha,1}-[E]_{\alpha,2})/([E]_{\alpha,1}+[E]_{\alpha,2})$, since these are not affected by a rescaling of the protein levels. As a consequence, it is possible to set some of these parameters to some reference values (e.g. $J_{P,1}+J_{P,2}=2$) without any loss of generality.

(**Section S1.2**) We show that any optimal solution to the enzyme minimization problem corresponds to the solution to an optimization problem in which the production fluxes are maximized, subject to a cap on the maximum enzyme levels. This means that our model captures two distinct selective pressures (flux maximization and enzyme economy) at the same time.

S1.1 - Family of solutions with constant metabolite concentrations

It is useful to introduce a compact notation for describing all the relevant variables and parameters in the model.

- $[m]_{\text{in}}$ indicates the concentration of any intracellular metabolite;
- $[m]_{\text{out}}$ indicates the concentration of any extracellular metabolite;
- $[E]$ indicates the concentration of any enzyme;
- $x=([m]_{\text{in}},[m]_{\text{out}},[E])$ is a vector containing all variables in the model;
- V indicates any intracellular flux;
- U indicates any transport flux;
- J indicates any demand flux;
- D indicates any permeability constant.

Since we won't consider in the following analysis any other kinetic parameter or the relative populations of the two bacterial species, so that there is no need to consider them explicitly. The kinetic equations in Eq. (1-3) can be then written generically as

$$V=[E]f([m]_{\text{in}}) \quad , \quad U=D([m]_{\text{in}}-[m]_{\text{out}}) \qquad \textbf{(Eq. S1)}$$

where $f([m]_{\text{in}})$ is a function describing the All mass-balance constraints (Eq. 4 from the main text) are linear constraints, either equalities or inequalities, of the fluxes. In fact, the only non-linear terms in the constraints are those involving the internal metabolites. Let us now suppose that $x^*=([m]_{\text{in}}^*,[m]_{\text{out}}^*,[E]^*)$ solves the mass-balance constraints with demand fluxes J and membrane permeabilities D . It is easy to check that, for any positive constant a , the vector $x^*(a)$ obtained by multiplying the enzyme concentrations by this constant, so that $x^*(a)=([m]_{\text{in}}^*,[m]_{\text{out}}^*,a[E]^*)$, solves the mass-balance equations (Main Text, Eq. 4), as long as the parameters J and D are multiplied by the same constant.

This relation relates a family of different models obtained by jointly varying the demand fluxes and the permeabilities constant. In the approach assumed in the main text, the cost function is a linear function of the enzyme concentrations, $C\propto[E]$; therefore, as the parameter a is varied, optimal solutions are mapped into optimal solutions, because the cost function is only multiplied by a constant and therefore its minima are not affected by the rescaling. This property allows us fix an

absolute scale for the J and D parameters, without losing any generality; indeed, we fixed the sum of the intracellular demand fluxes $J_{P,1}+J_{P,2}=2$. As an application of this relation, consider Fig. 2 in the main text, which is obtained for $J_{P,1}=J_{P,2}=1$ and $J_{P,0}=0$. If we had set the demand fluxes to $J_{P,1}=J_{P,2}=a$ with $a>0$, one would have obtained exactly the same protein asymmetry landscape, with the only difference that the axes would have been rescaled by a factor $1/a$.

S1.2 - Flux maximization and enzyme level minimization are dual problems

In our work we focused on enzyme concentration minimization, subject to flux constraints ("demand fluxes"). In this section we will show that this approach yields the same results as the maximization of the biosynthetic fluxes with a cap on the total concentration of enzymes. The demand fluxes constrain the biosynthetic fluxes and the P metabolite excretion as:

$$V_{P,1}\geq J_{P,1} \ , \quad V_{P,2}\geq J_{P,2} \ , \quad n_1 U_{P,1}+n_2 U_{P,2}\geq J_{P,0} \qquad \textbf{(Eq. S2)}$$

By introducing the total excretion rate $V_{P,0}\equiv n_1 U_{P,1}+n_2 U_{P,2}$ we can write these constraints in a compact form as:

$$V_{P,i}\geq J_{P,i} \ , \quad i=1,2,3 \qquad \textbf{(Eq. S2)}$$

The optimization problem can be recasted as:

$$\min_x C(x) \ \text{s.t.} \ V_{P,i}(x)\geq J_{P,i} \ , \quad i=1,2,3 \qquad \textbf{(Eq. S3)}$$

where x , as before, stands for the set of metabolite and enzyme concentrations. The additional constraints on the variables due to the mass balance equations do not play any role in the following, and thus we are not writing them explicitly in Eq. (S3). Let us call x^* the solution of this optimization problem; similarly, $C^*=C(x^*)$ and $V^*_{P,i}=V_{P,i}(x^*)$. In our simulations, the constraints in Eq. (S3) are satisfied by the optimal solution with equalities, $V^*_{P,i}=J_{P,i}$. This is a reasonable results, since one expects that minimum enzyme concentration needed to sustain the demand fluxes should increase along with the fluxes. This request is expressed in mathematical terms by the strict inequality $\mathrm{d}C^*/\mathrm{d}J_{P,i}>0$ (the shadow price of the constraint has to be positive) If this inequality is satisfied, the constraints said to be active, and $V^*_{P,i}=J_{P,i}$. Let us now consider the following "auxiliary" problem:

$$\min_{x,y} C(x) \ \text{s.t.} \ y\geq y^* \ , \ V_{P,i}(x)=y J_{P,i} \ , \quad i=1,2,3 \qquad \textbf{(Eq. S4)}$$

Here, we introduced an auxiliary variable y which is constrained to be larger than some constant y^*. It is easy to check that if $y^*=1$, then a solution of Eq. (S3) satisfies the constraints of Eq. (S4) and vice-versa; but it is also easy to see that an optimal solution of one of the two problems provides an optimal solution to the other one. This follows from the fact that the solution space (the set of all possible vectors x satisfying the constraints) of the auxiliary problem is a subset of the original one, and contains its optimal solution (see Fig. N1A). If the solution of the two problems were different, we would obtain a contradiction. Therefore, the “protein minimization” problem and the “auxiliary” problem are completely equivalent. But then, let us consider the following “flux maximization” problem:

$$\max_{x,y} y \text{ s.t. } C(x) \le C^* , \quad V_{P,i}(x) = y J_{P,i} , \quad i=1,2,3 \qquad \textbf{(Eq. S5)}$$

Again, it is easy to see that this problem is equivalent to the auxiliary one by comparing the solution space of the two. Therefore, we have shown that the three problems are equivalent: in our model, minimizing the protein concentration with constraints on the demand fluxes is equivalent to maximizing the fluxes of the product metabolites, subject to a cap on the enzyme concentration.

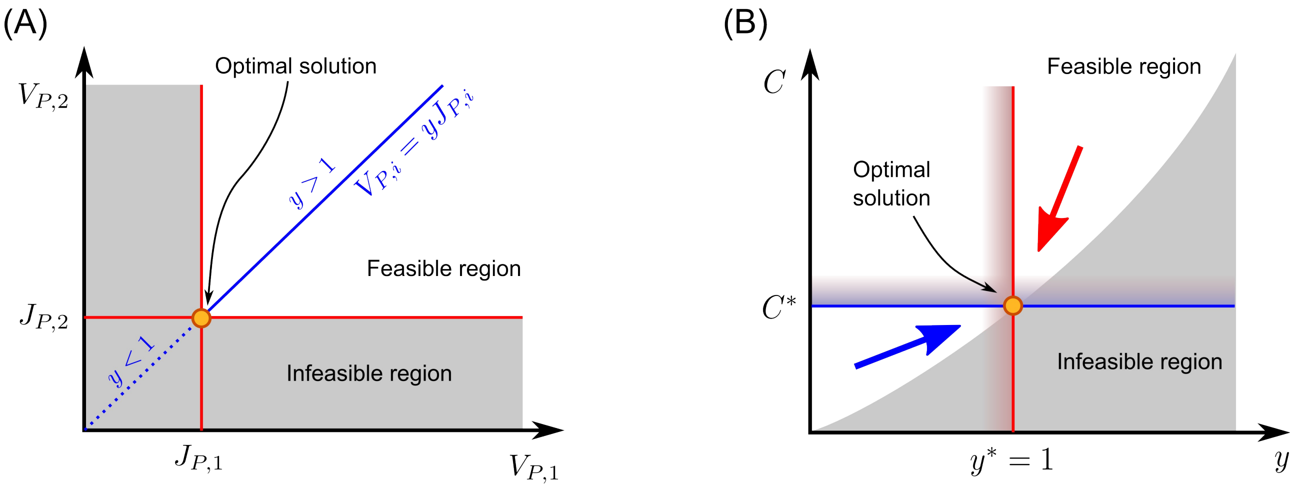

***Figure N1. (A) Equivalence of the solutions of the “protein minimization” problem (minimize** C s.t. $V_{P,i} \ge J_{P,i}$**) and the “auxiliary” problem (minimize** C s.t. $y \ge y^* = 1$ **and** $V_{P,i} = yJ_{P,i}$**).** *In the protein minimization problem the production fluxes $V_{P,i}$ are constrained to be larger than the demand fluxes $J_{P,i}$ (red lines); the condition $\mathrm{d}C^*/\mathrm{d}J_{P,i}>0$ implies that the optimal solution is found when $V_{P,i}=J_{P,i}$. In the auxiliary problem one still optimizes for the protein levels, but restricting the solution to the line $V_{P,i}=yJ_{P,i}$ (in blue) with $y \ge 1$. (B) Equivalence of the “auxiliary problem” (minimize C s.t. $y \ge y^*$) and the “flux maximization” problem (maximize y s.t. $C \le C^*$). The coloured arrow show the directions of the optimization problems; the same colors indicate the relevant constraints ($y \ge y^*$ and . $C \le C^*$). In this case the condition $\mathrm{d}C^*/\mathrm{d}J_{P,i}>0$ guarantees that the slope of the border between the “feasible” and the “infeasible” regions is positive.*

Supplementary Note S2: Sensitivity analysis procedure

In this note we resume the approach we adopt for sensitivity analyses performed to identify which parameters impact the most on the structure of the optimal solutions. First, we define $\{k_i\}$ as the set of parameters to be perturbed (e.g. the kinetic constants), and $\{x_i\}$ as the variables in the optimization problem (*i.e.* protein and metabolite concentrations). For each parameter k_i, a uniform probability distribution $p_i(k_i)$, centred around a reference value k_i^0, is defined. In order to identify which parameters are mostly involved in the transition from the symmetric to the asymmetric solution, we focused on a point close to the frontier separating the two regions with $A_S=0$ (symmetric solution) and $|A_S|=1$, namely $1/K_I=10$, $D_X=4$ and $D_P=5$ (see Main Text Fig. 2). Then, a set of $N=200$ different combinations of parameters $\{k_i\}_\alpha$, $\alpha=1,\ldots,N$ was generated, and for each set of parameters $\{k_i\}_\alpha$ we computed the optimal concentrations $\{x_i^*\}_\alpha$.

The optimization presents some additional challenges with respect to the other cases discussed in the manuscript, as one has to check the convergence of the minimization problems without relying on smoothness properties of the optimal solution. Furthermore, we chose a point on the frontier between the symmetric and the asymmetric region, where the cost function presents two almost-degenerate minima (i.e. two points in the concentration space where the cost function attains roughly the same value). In this condition, local optimization methods hardly converge to the correct minimum. For the sensitivity analysis, the following optimization method was used, each optimal solution was obtained from several minimization rounds, each one using a different (random) starting point or seed. The iterative procedure we followed is the following:

1. Set a counter $q=0$.
2. Increment the counter q by 1. For each set of parameters $\{k_i\}_\alpha$, $\alpha=1,\ldots,N$ we compute an optimal solution $\{x_i\}_\alpha^q$ starting the minimization algorithm from a random seed.
3. If $q=1$, set $\{x_i^*\}_\alpha=\{x_i\}_\alpha^q$. If instead $q>1$, compare the solutions $\{x_i\}_\alpha^q$ with the ones obtained at the previous step, $\{x_i\}_\alpha^{q-1}$. If the cost function evaluated with the latest solution is smaller than the cost function evaluated at the previous step, set $\{x_i^*\}_\alpha=\{x_i\}_\alpha^q$.
4. If, during the evaluation at step 3, none of the N sets of solutions $\{x_i^*\}_\alpha$ has changed, exit. Otherwise, go to step (2).

The fraction of solutions getting updated at each iterations decreases constantly, so that this

algorithm guarantees that the vast majority of the solutions $\{x_i^*\}_\alpha$ converge on the true optimal configuration. We end up with a dataset of parameters $\{k_i\}_\alpha$ and associated optimal solutions $\{x_i^*\}_\alpha$, $\alpha=1,\ldots,N$. Since the optimal concentrations depend on the chosen set of parameters, any function $f(x^*)$ of the optimal concentrations (*e.g.* the protein asymmetry A_S, or the cost function itself) is a random variable itself. In particular, we are interested in checking which parameters most affect the protein asymmetry, which is clearly signalled by the value of A_S).

Supplementary figures and tables caption

Supplementary Figures

Supplementary Figure S1. Protein asymmetry A_α in the case of competitive inhibition of P on the E_S enzyme. *The plots show the protein asymmetry A_α, for four different values of the inhibition constant (increasing inhibition, from left to right: $1/K_I = 3, 10, 30, 100$) for the case of competitive inhibition. In this case we set $S_{max} = 10$. In absence of any kind of inhibition ($1/K_I \to 0$) the optimal solution is always symmetric, i.e., $A_\alpha = 0$ for all proteins. An asymmetric solution emerges when inhibition is relevant and D_X/D_P is large enough. The darker color in the $1/K_I = 100$ case highlights the region in which $[E_{S,2}] = [E_{X,1}] = 0$, and the pathway is completely split between the two cell types. The case of non-competitive inhibition is shown in Fig. 2.*

Supplementary Figure S2. Optimal solutions as a function of the external P demand flux $J_{P,0}$, for a fixed value of the internal demand fluxes. *Top panels (A-D) show the optimal solution (in red), together with particular solutions obtained by forcing to zero the levels of particular enzymes ($E_{S,2}$ and $E_{X,1}$). The bottom panels (E-H) show the absolute protein levels in the optimal solution. We used the following settings: for all the cases the demand of the cells are $J_{P,1} = J_{p,2} = 1$; then (A,E) $K_I = 0$, $D_X = 2$, $D_P = 2$; (B,F) $K_I = 1/25$, $D_X = 2$, $D_P = 2$; (C,G) $K_I = 1/25$, $D_X = 10$, $D_P = 10$; (D,H) $K_I = 1/25$, $D_X = 20$, $D_P = 4$. In each of the bottom plots, the protein levels are normalized to the maximum level attained by any of the six proteins at any value of $J_{P,0}$, that is, $\epsilon_{\alpha,i}(J_{P,0}) = E_{\alpha,i}(J_{P,0}) / [\max_{(J_{P,0},\alpha,i)} E_{\alpha,i}(J_{P,0})]$. For the case of asymmetric solution, only those with protein asymmetry $A_S \geq 0$ are shown.*

Supplementary Figure S3. Optimal solutions as a function of demand flux asymmetry $\rho_J = (J_{P,1} - J_{P,2})/(J_{P,1} + J_{p,2})$, for a fixed value of the total flux, $J_{P,1} + J_{p,2} = 2$. *Top panels (A-D) show the optimal solution (in red), together with particular solutions obtained by forcing to zero the levels of specific enzymes ($E_{S,1}$, $E_{S,2}$ and $E_{X,2}$). The bottom panels (E-H) show the absolute enzymes concentration in the optimal solution. We used the following settings (same as Fig. S2, using $J_{P,0} = 0$): (A, E) $K_I = 0$, $D_X = 2$, $D_P = 2$; (B, F) $K_I = 1/25$, $D_X = 2$, $D_P = 2$; (C, G) $K_I = 1/25$, $D_X = 10$, $D_P = 10$; (D, H) $K_I = 1/25$, $D_X = 20$, $D_P = 4$. In each of the bottom plots, the enzyme concentrations are normalized as in Figure S2. Note that when ρ_J is different from zero, the symmetry between the two optimal solutions with $A_S > 0$ and $A_X < 0$ is broken, so that we have to consider them separately. In particular, the solution with $A_S < 0$ ($[E_{S,1}] = 0$) is optimal when ρ_J >0.*

Supplementary Figure S4. Optimal solutions as a function of the relative population size n_1. *As in Figure S3, the two solutions with $A_S = -1$ ($[E_{S,2}] = 0$) and $A_S = 1$ ($[E_{S,1}] = 0$) are no longer equivalent when n_1 is different than 0.5. Top panels (A-D) show the optimal solution (in red), together with particular solutions obtained by forcing to zero the levels of specific enzymes ($E_{S,1}$, $E_{S,2}$ and $E_{X,2}$). Bottom panels (E-H) show the absolute enzyme concentrations in the optimal solutions; the enzyme concentrations are normalized as in Figure S2. We used the following settings: (A, E) $K_I = 1/10$, $D_X = 1.5$, $D_P = 1.5$, $J_{P,0} = 0$; (B, F) $K_I = 1/10$, $D_X = 5$, $D_P = 3$, $J_{P,0} = 0$; (C, G) $K_I = 1/20$, $D_X = 10$, $D_P = 10$, $J_{P,0} = 0$; (D, H) $K_I = 1/25$, $D_X = 10$, $D_P = 10$, $J_{P,0} = 1$.*

Supplementary Figure S5. Solutions obtained from the extended model with different numbers of permeable metabolites. *Metabolites which are allowed to efficiently cross the cell membrane: 1, 2 or 5 (panels A, B and C, respectively). All other settings are the same as in Main Figure 4. When more than one metabolite (other than the "product" metabolite) are allowed to be exchanged across the different bacterial*

cells, the pathways are not neatly divided across the two cells, as in panel A; instead, the optimal enzyme levels change gradually along the pathway (panels B and C).

Supplementary Figure S6. Multiple sequences alignment (MSA) of the trpE gene in twelve different strains of B. aphidicola, together with the corresponding homologs in E. coli K12 and S. marcescens. *All the sequences are about the same size (~515 residues) and the figure is focused on the region corresponding to the allosteric binding site. The first, second an third rows, from top to bottom, correspond to the sequences of S. marcesens, E. coli and B. aphidicola (C. cedri) respectively. Arrow at at columns 21 and 40 indicate the key residues involved in the allosteric inhibition mechanism, according to [1]–[3]. Is worth to note, the the substitution Ser40→Thr40 present in some of the strains may not imply a big deal since both residues have similar physicochemical properties.*

Supplementary Tables

Supplementary Table S1. Results of the sensitivity analysis performed over the kinetic parameters on the model with uncompetitive inhibition. *The table include two sheets: in the first one ("ES asymmetry") we show the results of the sensitivity analysis respect protein asymmetry, whereas in the second sheet ("Total proteins") the same sensitivity analysis was repeated with respect to the sum of the protein concentrations of both bacterial species. Results for the former case are explained in the Main Text. In the latter case, an increase in turnover numbers or in the substrate concentration* $[S]$ *reduces the minimum amount of proteins needed to sustain the product flux; Conversely, this minimum protein level increases when the demand fluxes or the amount of inhibition are increased.*

Supplementary Table S2. Physicochemical properties and rule-based estimators to evaluate membrane permeability of the metabolites involved in the biosynthesis of aromatic and branched chain amino acid. *The table include data from size different pathways. Columns abbreviation: molecular weight (MW); hydrogen bond donor (HBD); hydrogen bond acceptor (HBA); Lipinsky rule of five (Le); Lipinsky rule of five extended (L5e); topological polar surface area (TPSA); 1Prule and 3PRule correspond to the two rule-based classifiers. sing these physicochemical parameters, we compute the Lipinski rule of five [4] as well as its extended version [5]. which allow us to discard compounds that violate more than one rule. Finally, we also adopted rule-based criteria proposed by Pham-The et al. [6] to classify compound permeability in High (H) Medium High (MH), Medium (M), Medium Low (ML) and Low (L). For instance, we used the 1PRule which classify compound permeability using the PSA, as well the 3PRule which combines molecular weight, PSA and LogD, for which we used AlogP as an estimator.*

Supplementary References

[1] J.-H. Kwak, K.-W. Hong, S.-H. Lee, J.-H. Hong, and S.-Y. Lee, "Identification of Amino Acid Residues Involved in Feedback Inhibition of the Anthranilate Synthase in Escherichia coli," *BMB Rep.*, vol. 32, no. 1, pp. 20–24, 1999.

[2] X. F. Tang, S. Ezaki, H. Atomi, and T. Imanaka, "Anthranilate synthase without an LLES motif from a hyperthermophilic archaeon is inhibited by tryptophan.," *Biochem. Biophys. Res. Commun.*, vol. 281, no. 4, pp. 858–65, Mar. 2001.

[3] M. G. Caligiuri and R. Bauerle, "Identification of amino acid residues involved in feedback

regulation of the anthranilate synthase complex from Salmonella typhimurium. Evidence for an amino-terminal regulatory site.," *J. Biol. Chem.*, vol. 266, no. 13, pp. 8328–35, May 1991.

[4] C. A. Lipinski, F. Lombardo, B. W. Dominy, and P. J. Feeney, "Experimental and computational approaches to estimate solubility and permeability in drug discovery and development settings.," *Adv. Drug Deliv. Rev.*, vol. 46, no. 1–3, pp. 3–26, Mar. 2001.

[5] D. F. Veber, S. R. Johnson, H.-Y. Cheng, B. R. Smith, K. W. Ward, and K. D. Kopple, "Molecular properties that influence the oral bioavailability of drug candidates.," *J. Med. Chem.*, vol. 45, no. 12, pp. 2615–23, Jun. 2002.

[6] H. Pham-The, I. González-Álvarez, M. Bermejo, T. Garrigues, H. Le-Thi-Thu, and M. Á. Cabrera-Pérez, "The Use of Rule-Based and QSPR Approaches in ADME Profiling: A Case Study on Caco-2 Permeability," *Mol. Inform.*, vol. 32, no. 5–6, pp. 459–479, Jun. 2013.
