# Supplementary figures and images for: Metabolic Complementation in Bacterial Communities: Necessary Conditions and Optimality
Source: Front Microbiol. 2016 Oct 7;7:1553. doi: 10.3389/fmicb.2016.01553 (PMC5054487; doi:10.3389/fmicb.2016.01553)

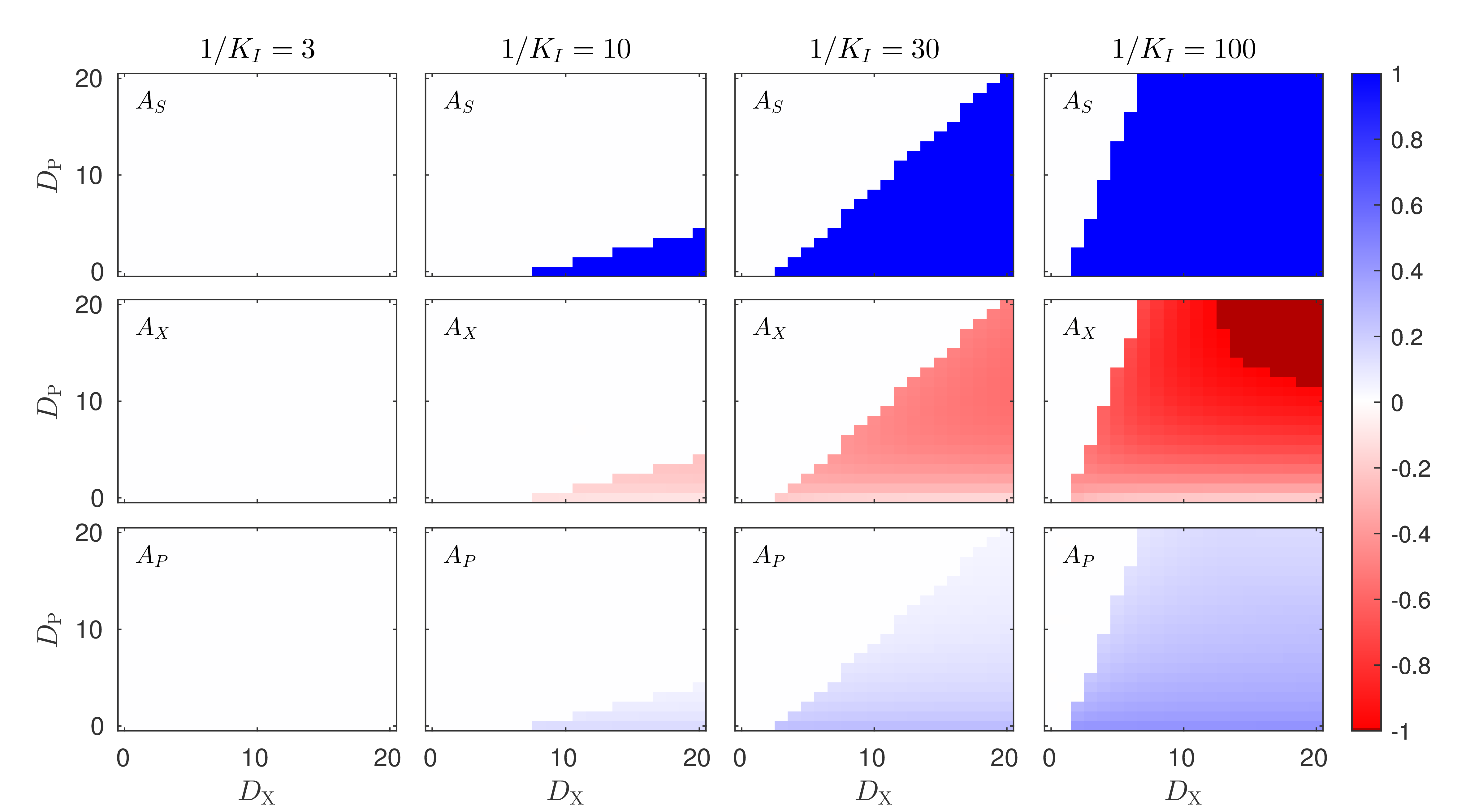

Supplement: Supplementary file 5 [file Image1.PNG]

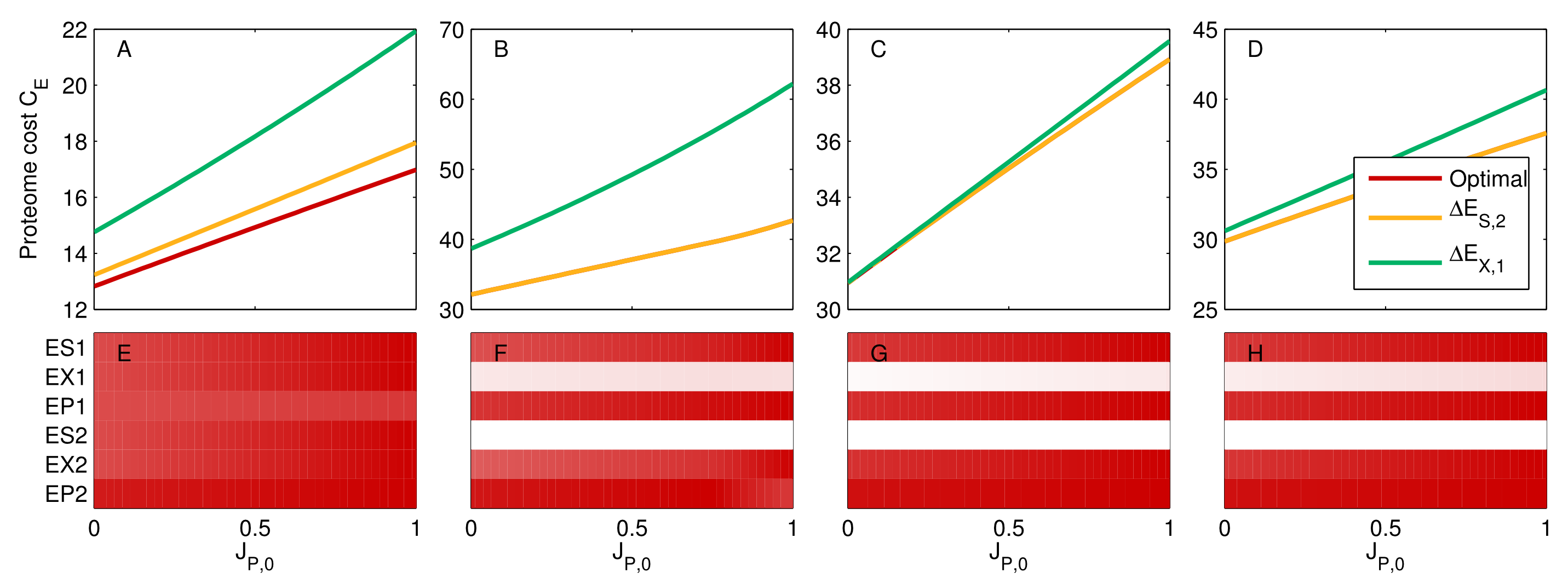

Supplement: Supplementary file 6 [file Image2.PNG]

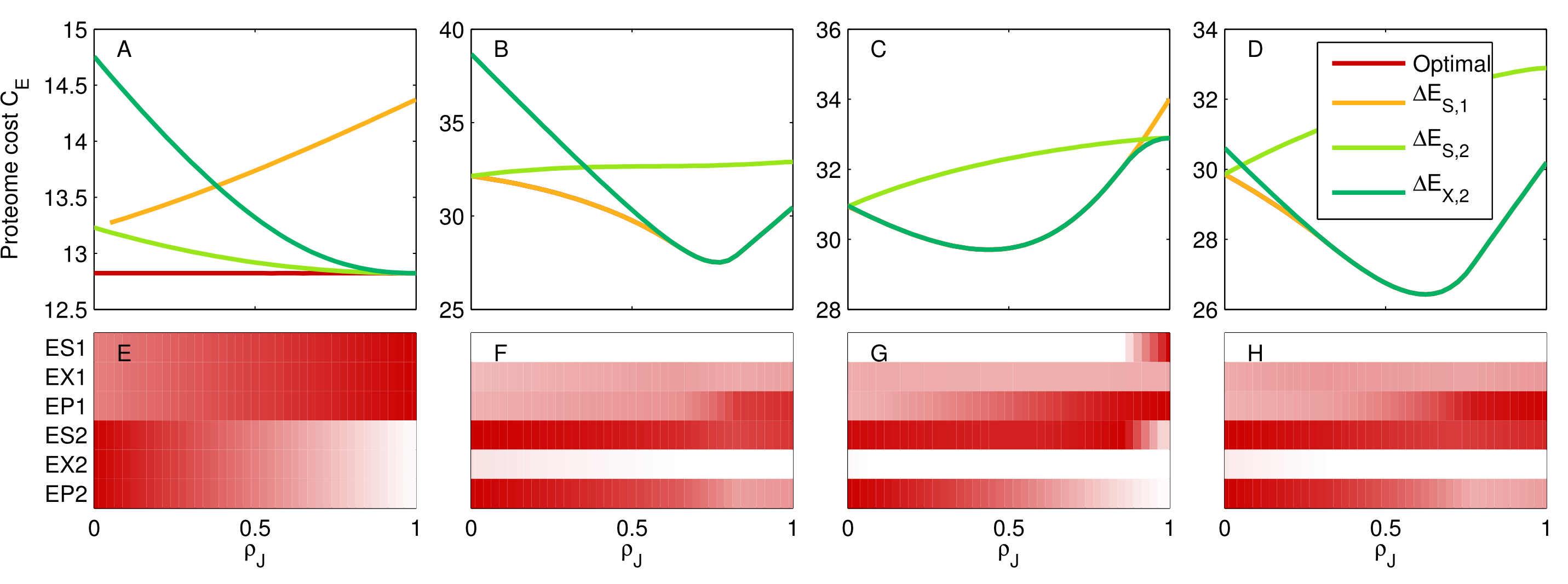

Supplement: Supplementary file 7 [file Image3.PNG]

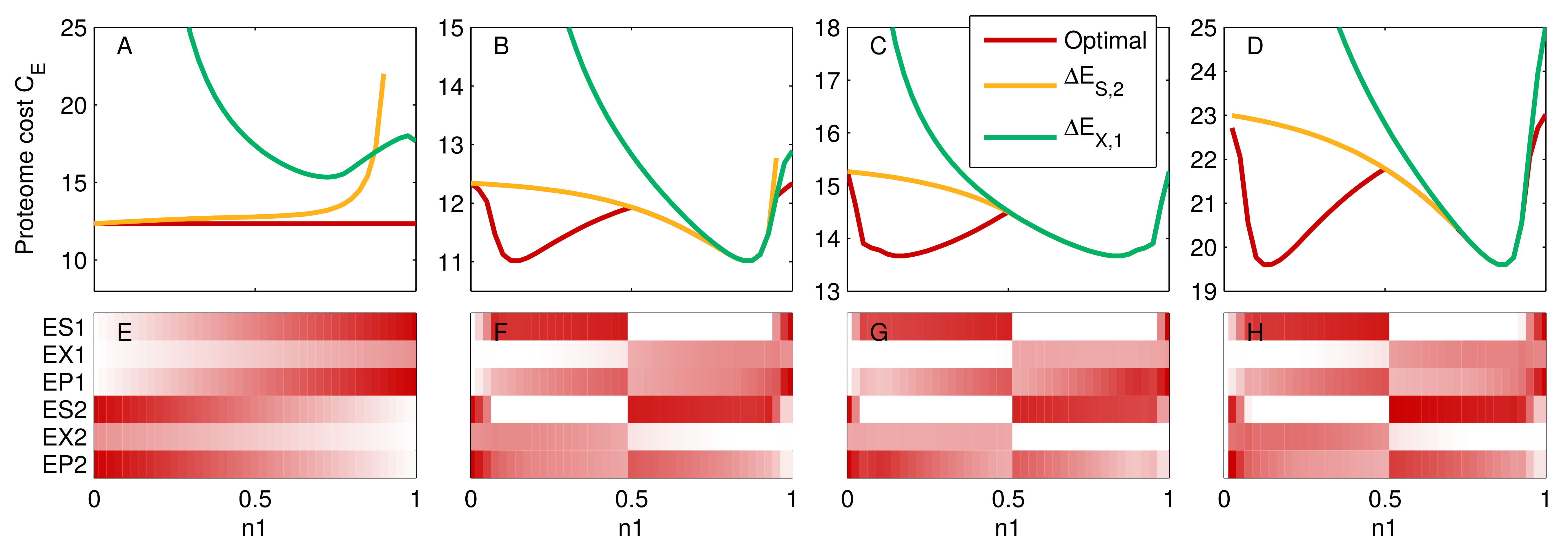

Supplement: Supplementary file 8 [file Image4.PNG]

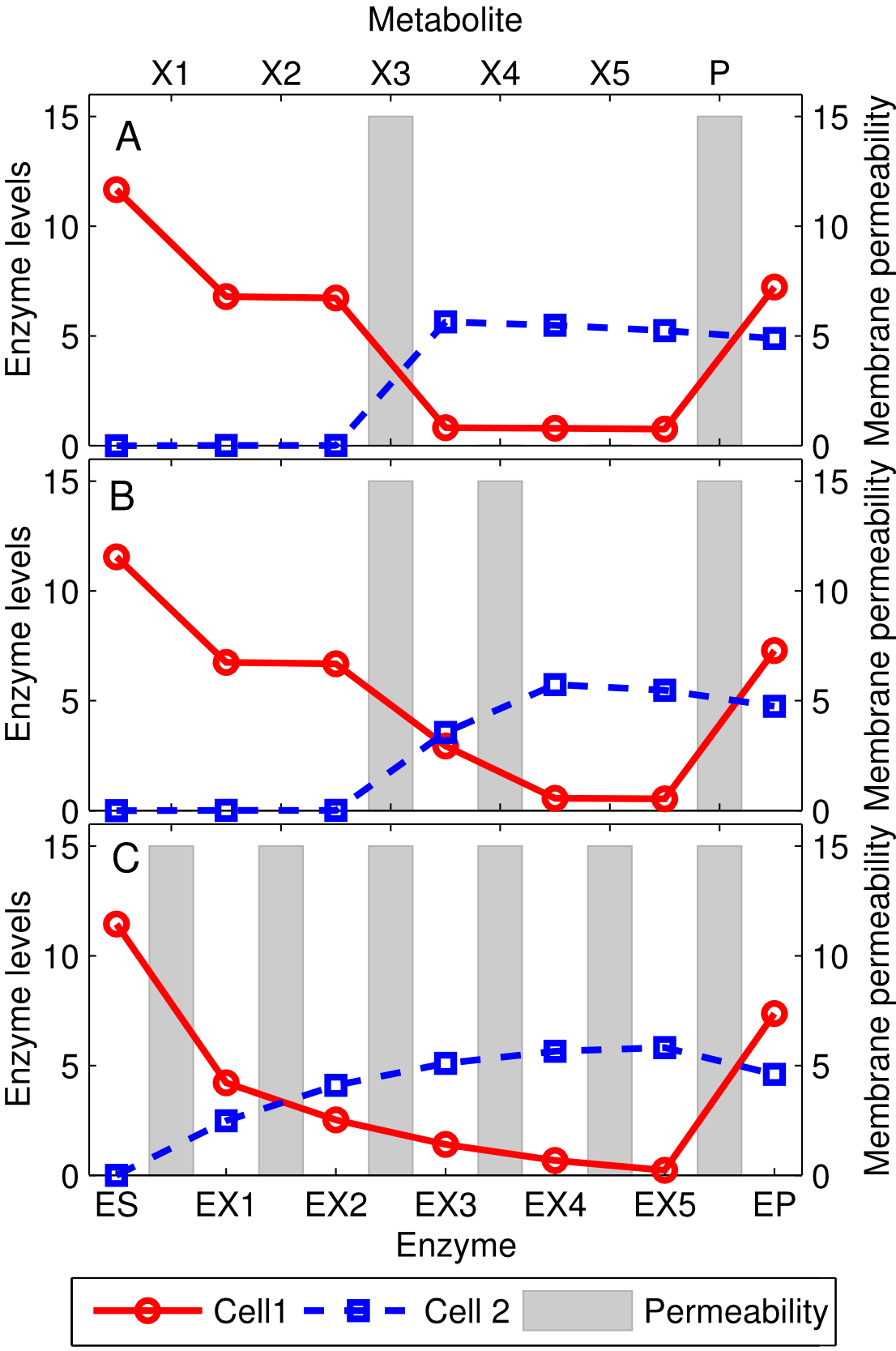

Supplement: Supplementary file 9 [file Image5.PNG]

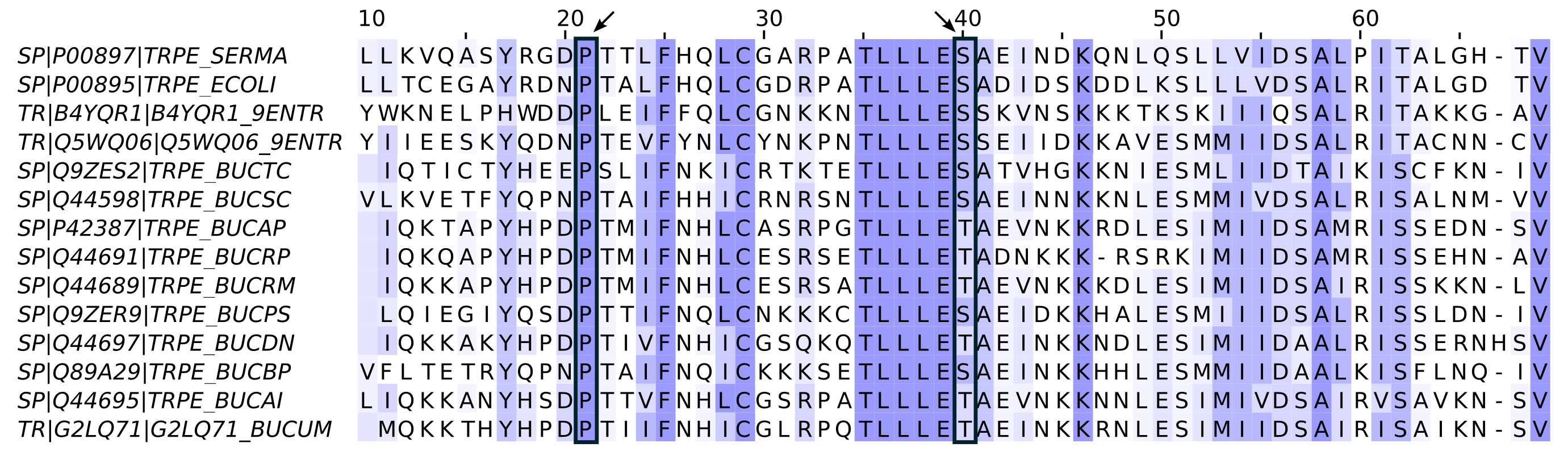

Supplement: Supplementary file 10 [file Image6.PNG]
